# A Case of Multifocal Primary Pyomyositis Complicated With Toxic Shock Syndrome in a Non-tropical Region

**DOI:** 10.7759/cureus.70446

**Published:** 2024-09-29

**Authors:** João R Corrêa, Ana P Silva, Maria J Pacheco, Renato Gonçalves, Dália Estevão

**Affiliations:** 1 Internal Medicine, Centro Hospitalar e Universitário Cova da Beira, Covilhã, PRT; 2 Rheumatology, Centro Hospitalar e Universitário Cova da Beira, Covilhã, PRT

**Keywords:** group a β-hemolytic streptococcus pyogenes, multifocal pyomyositis, primary pyomyositis, toxic shock syndrome (tss), tropical pyomyositis

## Abstract

Primary pyomyositis, also known as tropical pyomyositis, is a primary bacterial infection of skeletal muscle following hematogenous infections. It is primarily caused by *Staphylococcus aureus* or Group A *Streptococcus* and predominantly affects children and young adults. Although rarely observed in temperate climates, its prevalence appears to be increasing. Here, we present the case of a 36-year-old male patient who manifested with persistent fever and inflammatory signs in multiple skeletal muscle locations following acute pharyngitis, further complicated by toxic shock syndrome within 48 h of admission. The blood cultures were positive for *Streptococcus pyogenes* and ultrasound evaluation demonstrated muscle tissue heterogeneity, associated with areas of liquid collection and subcutaneous edema, in the right pectoral muscles and bilaterally in the fibularis longus and extensor digitorum longus muscles, confirming the diagnosis of primary pyomyositis. After treatment with a prolonged course of antibiotics, the patient showed substantial clinical improvement and was completely asymptomatic at 6-month follow-up. This case illustrates the possible risks associated with primary pyomyositis and the importance of its early recognition and treatment, regardless of geographic location.

## Introduction

Primary pyomyositis is a purulent infection of skeletal muscle that arises from hematogenous infection, without the presence of penetrating trauma or contiguous infection [1].

Primary pyomyositis affects mostly children and young adults and because of its historical link to tropical regions, where it remains more prevalent, is also known as “Tropical Myositis” [2]. Despite remaining a rare disease in temperate climates, responsible for less than 0.01% of hospital admissions, its prevalence in non-tropical regions is rising [3,4].

The most common pathogens involved are Staphylococcus aureus (responsible for more than 75% of the cases) and Group A Streptococcus (responsible for up to 20% of the cases) [5,6].

## Case presentation

A 36-year-old male patient was evaluated in our emergency department (ED) for persistent fever, for the past 7 days, bilateral painful leg swelling, and painful mobilization of the right arm, in the last 48 hours (h). He also reported dry cough and odynophagia for the past 7 days, slightly improved at the time of observation, with no other symptoms.

The patient migrated from Brazil 1 year before with no other trips occurring since. He had no past history of known diseases or prior medication use and reported no high-risk sexual behavior.

The physical examination demonstrated the presence of fever (temperature of 39.1°C), tonsillar hypertrophy, and oropharyngeal hyperemia without the presence of pus, tenderness with inflammatory signs on the right superior pectoral region, and bilateral tenderness in the calf region.

Initial laboratory workup (Table 1) showed a marked elevation of the inflammatory parameters - leukocytosis (27.6 × 10^3^/µL) with neutrophil predominance, elevated C-reactive protein (51.31 mg/dL), procalcitonin (26.09 ng/mL) and ferritin (3709.2 ng/mL) levels and an elevated erythrocyte sedimentation rate (125 mm/H) - creatinine kinase and myoglobin elevation (1174 U/L and 932 ng/mL, respectively), thrombocytopenia (90 × 10^3^/µL), hyperbilirubinemia (4.02 mg/dL) and elevation of aspartate transaminase (92 U/L), alanine transaminase (150 U/L), and gamma-glutamyl transferase (478 U/L).

**Table 1:** Laboratory values on admission

Laboratory Parameter	Value (Reference Range)	Units
White blood cell count	27.6 (4.0 – 10.0) × 10^3^	Cells/microliter
Neutrophils	25.6 (1.5 – 8.0) × 10^3^	Cells/microliter
Lymphocytes	1.1 (0.8 – 4.0) × 10^3^	Cells/microliter
Hemoglobin	15.1 (13.6 – 18.0)	g/dL
Platelets	90 (150 – 450) × 10^3^	Cells/microliter
Creatinine	1.11 (0.70 – 1.20)	mg/dL
Blood urea nitrogen	15 (8 – 22)	mg/dL
Aspartate transaminase	92 (0 – 38)	U/L
Alanine transaminase	150 (0 – 41)	U/L
Gamma-glutamyl transferase	478 (7 – 66)	U/L
Bilirubin	4.02 (0.00 – 1.10)	mg/dL
Creatinine kinase	1174 (39 – 308)	U/L
Myoglobin	932 (≤ 154.9)	ng/dL
C-reactive protein	51.31 (0.00 – 0.50)	mg/dL
Procalcitonin	26.09 (< 0.05)	ng/mL
Ferritin	3709.2 (30.0 – 400.0)	ng/mL
Erythrocyte sedimentation rate	125 (0 – 15)	mm/H

The rapid detection test for both *Streptococcus *group A and methicillin-resistant *S. aureus* was negative, but blood cultures were positive within 48h for *Streptococcus pyogenes* sensitive to penicillin, vancomycin, clindamycin, and trimethoprim-sulfamethoxazole.

**Figure 1:**
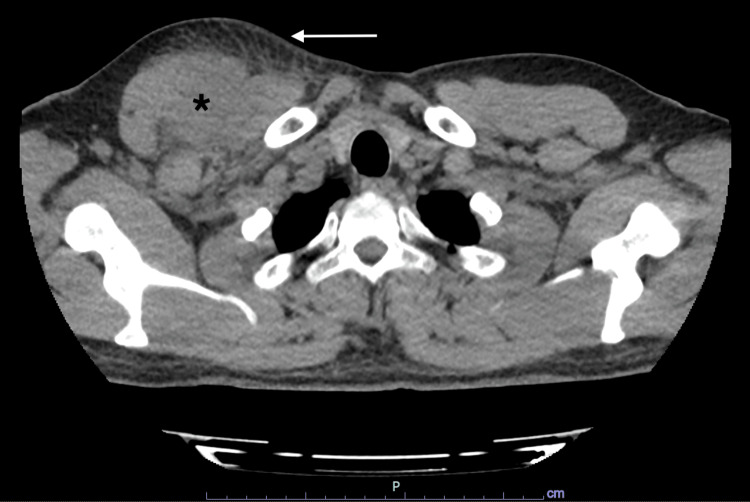
Thoracic CT scan exhibiting asymmetric and heterogeneous hypertrophy in the right pectoral muscle (asterisk) and adjacent subcutaneous tissue densification (arrow)

Initial thoracic computed tomography (CT) scan in the ED exhibited an asymmetric and heterogeneous hypertrophy in the right pectoral muscle associated with densification in the adjacent subcutaneous tissue (Figure 1).

**Figure 2:**
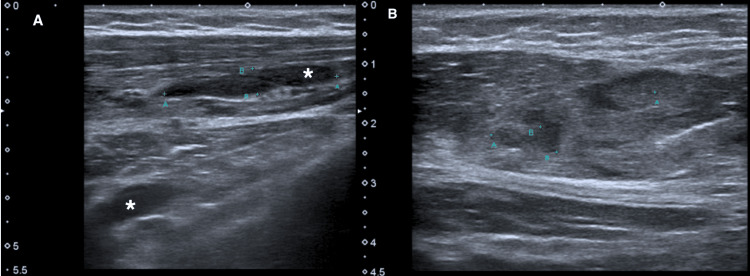
Ultrasound of the pectoral muscles, different views, showing multiple areas of liquid collection (asterisk) on panel A and muscle tissue heterogeneity on panel B

**Figure 3:**
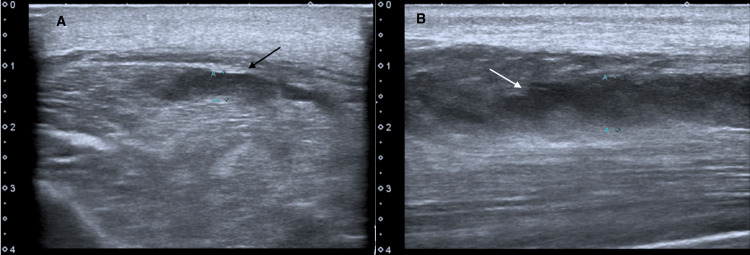
Transverse (Panel A) and longitudinal views (Panel B) of the right leg ultrasound showing muscle tissue heterogeneity and areas of liquid collection (arrows)

Ultrasound evaluation of the pectoral muscles later revealed extensive and diffuse muscle tissue heterogeneity and the presence of multiple areas of liquid collection, on the right side, associated with subcutaneous tissue edema, confirming the diagnosis of myositis (Figure 2). Bilateral leg ultrasound imaging had the same findings along the fibularis longus muscle and extensor digitorum longus muscle (Figure 3).

The patient was initially treated with ceftriaxone and admitted to hospital care. At the 48-hour mark (Table 2), due to worsening of the inflammatory markers, thrombocytopenia and hyperbilirubinemia and, de novo, hypoxemia and acute kidney injury, antibiotic therapy was changed to piperacillin-tazobactam and clindamycin, and the patient admitted into the intensive care unit (ICU) for closer monitoring.

**Table 2:** Laboratory values 48 h after admission

Laboratory Parameter	Value (Reference Range)	Units
White blood cell count	24.4 (4.0 – 10.0) × 10^3^	Cells/microliter
Neutrophils	22.2 (1.5 – 8.0) × 10^3^	Cells/microliter
Lymphocytes	1.1 (0.8 – 4.0) × 10^3^	Cells/microliter
Hemoglobin	13.0 (13.6 – 18.0)	g/dL
Platelets	80 (150 – 450) × 10^3^	Cells/microliter
Creatinine	2.58 (0.70 – 1.20)	mg/dL
Blood urea nitrogen	73 (8 – 22)	mg/dL
Aspartate transaminase	84 (0 – 38)	U/L
Alanine transaminase	98 (0 – 41)	U/L
Gamma-glutamyl transferase	321 (7 – 66)	U/L
Bilirubin	6.80 (0.00 – 1.10)	mg/dL
C-reactive protein	62.19 (0.00 – 0.50)	mg/dL
Procalcitonin	29.24 (< 0.05)	ng/mL

After a 72-hour stay in the ICU, needing only oxygen therapy and intravenous (IV) fluids, the patient started showing signs of improvement and was transferred back to the medical ward where he maintained IV antibiotic therapy for a total of 6 weeks.

Neither transesophageal echocardiography nor vertebral and pelvis CT scans, performed during the hospital stay, showed signs of endocarditis or osteomyelitis, respectively. Both abdominal and articular ultrasounds were without abnormal findings as well. Thoracic angiographic CT scan and bronchoscopy were also normal and bronchoalveolar lavage without the presence of infection disease.

Blood cultures performed at 2 and 4 weeks were negative. Serologic markers for autoimmune disorders, chronic inflammatory conditions, and immune deficiencies were negative during admission and follow-up. Other concurrent infections, namely infection with the human immunodeficiency virus (HIV), hepatitis B or C, parvovirus B19, tuberculosis, leishmaniasis, toxoplasmosis, Lyme disease, rickettsiosis, leptospirosis or Q fever were excluded; the patient was immune to infection with cytomegalovirus, Epstein-Barr virus, herpes simplex virus, varicella-zoster virus and rubella virus.

At discharge, he maintained oral antibiotic therapy with amoxicillin-clavulanic acid for another 2 weeks and physical rehabilitation. After 6 months of follow-up, he was able to run and do weight lifting.

## Discussion

Primary pyomyositis generally affects young patients in whom a recent or concurrent infection is present [2]. It manifests as fever and pain, usually localized to a single muscle, although multifocal infection can occur in up to 20% of the cases [7,8]. The more common sites of infection are the lower limb muscles, muscles of the hip and pelvis, and upper limb muscles [5].

In this case, the patient in question manifested with a multifocal skeletal muscle infection, involving an atypical site (pectoral muscle), following acute pharyngitis due to *S. pyogenes* with hematologic dissemination. The presence of an underlying immunocompromising condition (such as HIV infection, diabetes mellitus, IV drug use, rheumatologic disorders, or malignancies), absent in our patient, increases the likelihood of primary pyomyositis and should be routinely checked [6,9].

In this patient, the clinical course was complicated by the presence of toxic shock syndrome. Septic arthritis, osteomyelitis, and septic pulmonary emboli are other possible complications. The risk of serious complications makes it particularly important to accurately and promptly diagnose and treat this condition [6,7].

Diagnosis requires radiographic imaging with magnetic resonance imaging (preferred method given the high sensitivity for the presence of muscle inflammation or abscess), ultrasound or CT scan, and microbiological confirmation with positive blood cultures or culture after abscess aspiration or muscle biopsy. Treatment should include drainage, if an abscess is present, and intravenous antibiotic therapy, guided by local susceptibility of the most frequent pathogens and adjusted after positive microbiological findings [1]. Broad-spectrum antibiotics are recommended in the presence of sepsis or an underlying immunocompromising condition, vancomycin or daptomycin should be used if methicillin-resistance is confirmed or suspected and clindamycin added in the case of toxic shock syndrome [1].

In our patient, given the presence of multiple liquid collections and positive response to antibiotic therapy, once it was changed to piperacillin-tazobactam plus clindamycin, we opted for a prolonged course of antibiotics instead - IV therapy until resolution of liquid collections and 2 weeks of oral therapy afterward.

## Conclusions

Considering the increasing prevalence of primary pyomyositis in non-tropical regions, all physicians should be familiar with this condition and clinical course. Given the risk of serious complications, such as toxic shock syndrome, a swift diagnosis, and treatment are crucial in primary pyomyositis. Empiric antibiotic therapy should cover the most common pathogens involved, according to local resistance rates, disease severity, and underlying immunocompromising conditions. If possible, drainage should be performed as well.
